# Perspectives of Refugee Youth Experiencing Homelessness: A Qualitative Study of Factors Impacting Mental Health and Resilience

**DOI:** 10.3389/fpsyt.2022.917200

**Published:** 2022-06-06

**Authors:** Bushra M. Khan, Jordana Waserman, Mitesh Patel

**Affiliations:** ^1^Department of Psychiatry, University of Toronto, Toronto, ON, Canada; ^2^Centre for Addiction and Mental Health, Toronto, ON, Canada; ^3^Department of Psychiatry, University of British Columbia, Vancouver, BC, Canada; ^4^Inner City Health Associates, Unity Health Toronto, Toronto, ON, Canada

**Keywords:** youth, refugee, migrant, homelessness, mental health

## Abstract

Homeless refugee youth experience high rates of traumatic and adverse experiences, a significant burden of mental health needs, and compounded barriers in accessing support services. Despite this, there is a paucity of literature exploring the unique intersections and vulnerabilities faced by this subgroup. This study of Youth Without Shelter (YWS), an emergency residence and referral agency serving homeless youth in Toronto, Canada was a qualitative study of homeless refugee youth. Research goals were to describe the mental health needs and identify the factors contributing to the resiliency of refugee youth experiencing homelessness. Data was collected through individual semi-structured interviews with YWS youth (*n* = 6) and analyzed using inductive thematic analysis with a realist lens. Results highlighted that the mental health of refugee youth experiencing homelessness was subject to system-level factors such as the immigration process (*n* = 6), housing insecurity (*n* = 5), finances (*n* = 5), education (*n* = 6), employment (*n* = 6) and sense of safety (*n* = 4), with acculturative stress, including culture shock (*n* = 3) and language (*n* = 4), also have negative effects. Goal directedness (*n* = 5), independence or responsibility (*n* = 4), and nurturing social connectedness with family (*n* = 5), peers (*n* = 6), and community (*n* = 4) contributed to participants' resilience. A model for planning future interventions was proposed and was informed by the lived experience of participants. This model focuses on initially on immediate and basic needs and reflected evidence gathered through this work to attend to long-term needs once individuals have integrated into society. Future efforts will be directed toward translating the lived experience of this population and developing practice guidelines to optimize care.

## Introduction

At the end of 2019, there were ≈ 79.5 million forcibly displaced individuals worldwide (1). Of these, an estimated 30–34 million are children under 18 years of age (1). Canada plays a significant role in housing displaced peoples, having seen a combined total of 202,562 refugees and asylum seekers by the end of 2019 (2). Moreover, there has been a surge in asylum seekers crossing from the United States to Canada outside of regular border points since the 2017 change in U.S. government administration and associated immigration restrictions. At least 58,255 refugee claims were made in Canada by irregular border crossings between February 2017 and March 2020 (3).

The present study explores the mental health of homeless refugee youth in particular. Refugees as a group face an increased risk of housing insecurity and homelessness compared to the general population and even other newcomers, due to a combination of factors such as lack of documents, low or unstable incomes, discrimination by landlords, limited knowledge about the housing market and their rights and responsibilities, lack of contacts and separation from supports, and poor language skills (4). Compounding this, youth's experiences of homelessness may be exacerbated compared to that of adults by a lack of experience living independently, a paucity of resources to aid in attaining secure housing, and the simultaneous navigation of developmental changes (5). Moreover, the lived experiences of refugee youth can be uniquely challenging compared to those of older refugees, for reasons including separation from family, assumption of adult responsibilities at an early age, and exposure to trauma and adversity during key developmental periods (6).

Overall, newcomer youth may have unique reasons for entering situations of homelessness. For instance, in their survey of 74 homeless newcomer youth in Toronto, family conflict and abuse from family featured prominently as reasons why newcomer youth entered situations of homelessness (7). Homeless refugee youth endorse a high burden of mental health needs, as evidenced by a 26.7% rate of suicide attempts and a 25.9% rate of traumatic experiences among homeless newcomer youth sampled in Toronto (7).

The unique intersection at which homeless refugee youth exist warrants further investigation in order to inform how to best support their wellbeing and bolster their resilience. Multiple different models of refugee mental health have been proposed. For one, Li et al. (8) categorize post-migratory challenges as belonging to three groups: “socioeconomic stressors, social and interpersonal stressors, and stressors related to the asylum process and immigration policies.” Alternatively, a model published by Ellis et al. (6) proposes four core stressors that inform risk and resilience: trauma, acculturation, isolation, and resettlement (which includes basic, financial, healthcare and legal needs). A social ecological model by Miller and Rasmussen (9) underscores the contribution of ongoing environmental post-migratory stressors to the mental health of refugees. Finally, Silove's ADAPT model (10) proposes five pillars: safety/security, bonds/networks, justice, roles and identities, and existential meaning. Regardless of the framework to which one subscribes, it is evident that determinants of refugees' mental health are complex, multifactorial, and layered, and that socioeconomic, political and interpersonal needs factor heavily into the equation.

It has become clear that the mental health of refugees is informed not only by pre-migratory trauma, it is equally affected by post-migratory stressors [e.g., (6, 9)]. Chronic post-migratory stressors may mediate the effect of pre-migratory trauma on refugees' mental health, by depleting individuals' coping reserves and revised models are crucial to understanding stressors and resiliency factors (9).

The present study pertains to youth residing at Youth Without Shelter (YWS), an emergency residence for homeless youth in Toronto. Youth Without Shelter (YWS) is an emergency residence and referral agency in Toronto, Canada, serving homeless youth ages 16–24 years old. YWS is open 24 h per day and seven days a week and offers emergency and transitional shelter. Programming is also offered at YWS with the aim to permanently reduce youth homelessness by employing a strengths-based approach and trauma-informed care.

Our study aims were firstly, to describe the factors that impact the mental health of refugee youth participants, and secondly, to describe the factors that contribute to resilience among refugee youth participants. In undertaking this research, we also contributed to informing further practice guidelines at YWS.

## Methods

### Study Design

This study is a qualitative investigation of the mental health needs and factors contributing to the resilience of refugee youth residing at YWS. This study was approved by the Health Science Research Ethics Board at a large publicly funded university in Canada (protocol 35078).

### Eligibility

A broad definition of refugees was used for this study and was based on the legal definition from the United Nations High Commissioner for Refugees which stated “a person who is outside his/her country of nationality or habitual residence; has a well-founded fear of persecution because of his/her race, religion, nationality, membership in a particular social group or political opinion; and is unable or unwilling to avail himself/herself of the protection of that country, or to return there, for fear of persecution” (11). For this study, the definition of refugee was broadened to include refugee claimants or asylum seekers who had not yet had their claim approved by the Immigration and Review Board but had similar lived experience to refugee youth with approved claims to be included for eligibility.

Eligible youth were identified by their case managers at YWS, who then briefly explained the study to their youth clients at weekly meetings. If the youth was interested in participating in the study, the youth provided consent to be contacted by the research team in the future or to contact the team directly. Translation services were provided as appropriate.

During the informed consent process, it was reiterated that participants were free to participate or withdraw from the study. As a psychiatrist working at YWS was a lead investigator on this study, youth were assured that their care would remain unaffected if they chose to not participate. Participants provided written or verbal informed consent for participation in the study and each was formally documented by a research team member in writing. Participants were also asked to consent to audiotaping and if they declined, consent for note taking was obtained.

### Data Collection

Individual semi-structured interviews were conducted with refugee youth ages 16 to 24 (*n* = 6) from June 2018 to August 2018. Participants were sampled using a purposive approach until thematic saturation was obtained until themes were fully developed and no new themes emerged (12).

A 45-min semi-structured interview occurred exploring perceived mental health needs and resiliency factors (Appendix A). Interviews were conducted in private offices either at YWS or CAMH and location was selected based on participant preference. Baseline demographic information, including country of origin and time spent in Canada, was collected at the outset.

Psychological discomfort was mitigated in the interview protocol by explaining to all participants prior to the commencement of the interview that they were not required to comment on any topic that made them uncomfortable or upset. Participants were also provided with information regarding follow-up support. Furthermore, YWS staffs were available onsite while interviews were conducted for mental health support. Mental health services were also available through a collaboration with an inner-city family health team with psychiatric services provided by the senior author. Their investigator role was fully disclosed to all study participants and they were not involved in the interview process or direct thematic analysis. Participants were informed that their participation and responses would not affect their psychiatric care.

Interviews were later transcribed verbatim, and participants were de identified in transcripts and field notes to ensure confidentiality.

### Analysis

Youth interviews were analyzed according to the six-phased method of thematic analysis proposed by Braun and Clarke (13). Qualitative interview transcripts were reviewed to address the research questions using inductive thematic analysis with a realist lens using QSR International NVivo 12 software. Due to large file sizes of scanned documents and resultant slow software speed, certain notes and annotations were instead recorded using pen and paper.

Author (author initials) familiarized themselves with the data by reading all youth interviews thoroughly prior to commencing the coding process. Interviews were initially annotated or “pre-coded” [(14), p. 16–17] and ideas for potential codes and themes, notes on individual participants, reflexive thoughts, and rationales for decisions were documented throughout.

Notes and annotations from the pre-coding process were used to compile a list of early descriptive codes of semantic concepts (e.g., “family,” “education,” “employment). These codes were generally developed inductively (i.e., data-driven), although areas of interest remained informed by the two guiding research questions.

The mind map function *in NVivo* was used to categorize these codes and group them loosely according to a socio-ecological framework. The mind map was then converted to a hierarchical coding scheme *in NVivo* [(14), p. 9] and used to code the interview transcripts. Codes were modified, recategorized or added as the researcher progressed through the transcripts. The above process was completed separately for the youth interviews.

After coding was completed separately for the youth interviews, the codes generated for each subgroup were amalgamated and synthesized into one new coding tree. The coding tree was revised such that redundant codes were removed, codes were more appropriately categorized or named, and new codes for previously un-coded sections of text were added. This new coding tree was then applied uniformly across all youth interviews. Attribute coding was also employed to record participant demographic information [(14), p. 55–59]. Figures and visual representations of codes were used to facilitate the process of searching for themes.

Participant quotations informed the development of major themes and all members of the research team discussed and refined these themes through an iterative process. To ensure the findings were accurate, a checking process was completed with a YWS staff member; a member of the study population was not available to participate in the checking process at the time this paper was drafted.

## Results

The participant sample consisted of six youth (Table 1). The average age of youth participants was 21.3 years (SD 2.4 years), with all but one participant being in their 20s. Two-thirds of youth were currently unaccompanied by family in Canada, including one participant who was initially accompanied by family but subsequently separated. Half of the participants were refugee claimants awaiting their hearings while others were unable to describe their immigration status. Two-thirds of youth had resided in Canada for > 1 year, with the mean duration of residence being 3.7 years (SD 3.0 years).

**Table 1:** Demographic characteristics of YWS youth participants (*N* = 6).

**Characteristic**	**Value (%)**
Age (mean = 21.3 years, SD = 2.4 years)	
≥18 years old	5 (83%)
<18 years old	1 (17%)
Gender	
Female	1 (17%)
Male	3 (50%)
Other	2 (33%)
Number of Languages Spoken	
1	1 (17%)
2	2 (33%)
3	3 (50%)
Continent of Origin	
North America	2 (33%)
Africa	2 (33%)
Asia	2 (33%)
Immigration Status	
Refugee Claimant	3 (50%)
Permanent Resident	1 (17%)
Unknown	2 (33%)
Time in Canada (mean = 3.7 years, SD = 3.0 years)	
< 1 year	2 (33%)
> 1 year	4 (67%)
Current Accompaniment by Family	
Accompanied	2 (33%)
Unaccompanied	4 (67%)

Two broad themes emerged as central to the mental health of homeless refugee youth. The first was system level factors, which encompassed the immigration process (*n* = 6), housing insecurity (*n* = 5), finances (*n* = 5), education (*n* = 6), employment (*n* = 6) and sense of safety (*n* = 4), and the second was acculturative stress as a product of transition to Canada, which comprised of culture shock (*n* = 3) and language (*n* = 4). Two other themes emerged with respect to their resiliency: fostering a sense of control through goal directedness (*n* = 5) and independence and responsibility (*n* = 4), as well as social connectedness and belonging with family (*n* = 5), peers (*n* = 6), and community (*n* = 4).

### System-Level Factors Impacting Mental Health

Refugee youth experiencing homelessness described their mental health needs as central to their lived experience and prioritized these needs in their narratives. Youth Participants (YP) also described a number of YWS services being useful in meeting and managing these needs.

#### Immigration Process

Participants described the immigration process (*n* = 6) as a source of anxiety where asylum claims, long periods of waiting, and the possibility of having to return to their countries of origin impacted their mental health. One youth described being a refugee as a source of stress: “for me now, Iam still waiting for my court date, like that is stress. If you are not refuge you would not count that among your stress” (YP B1). Another youth described additional logistical issues: “every week I call my lawyer, I ask her if there is any updates. She says no. That makes me, like, worried” (YP B2). Waiting periods characterized the immigration processes, with postponements being common; as this participant described, they wanted resolution “really bad but I need to wait” (YP B1).

The fear of claim rejection and having to return to their country of origin was noted. This participant expressed their anxiety about being forced to leave Canada: “Iam feeling scared if I go back. Always thinking about it. If they did not accept me here, what should I do Every day I still thinking about it, every time” (YP B2). Legal status placed youth in vulnerable positions and allowed for the persistence of factors that caused increase stress: “if I apply for refugee and then I get denied. I would have to go back home So Iam just trying to take my time. I do not want to rush” (YP B5).

Youth participants described YWS being successful in mitigating stress associated with the immigration process through existing interventions. A YP testified to the positive impact of YWS' support with their application, reporting that “since my arrival to YWS, I got a lot of help with my refugee papers, which so feel that my papers are in safe hands” (YP B4).

#### Housing Insecurity

Housing insecurity (*n* = 5) was a prominent feature of youth mental health, as youth struggled with separation from family and the transition to living in a shelter. One participant spoke to dichotomous experiences of fear and comfort with the shelter:

“The emergency part is very intense. I did not like it. I was extremely stressed when I was there and scared from the possibility of being discharged which could happen after four warnings. But when I moved to the transitional part it was more relaxed. I can move freely. The curfew was longer. I can go up and down the stairs which was not allowed in the E. part. It feels like home.” (YP B4).

Another participant discussed the initial challenge they faced in adjusting to shared accommodation, and how their immigration concerns overshadowed this: “When you have big problems, you do not care where you live, There is bigger problems, which is my court, my acceptance.” (YP B2). Specifically, this youth had unique concerns about being discharged from YWS for behavioral reasons fostered increased anxiety because “if I make a mistake and Iam discharged, they are gonna send me, like, somewhere, like, I do not even know where I suppose to go” (YP B3). Graduating from YWS and finding housing was another source of concern: “housing it is so important. Here is good but I cannot live, you know.” (YP B2).

Several housing interventions are in place at YWS. Although not traditionally conceptualized as a mental health intervention, these were experienced by youth as improving mental wellbeing, as “for now, staying in the shelter freely is support” (YP B1). Other YWS housing centered interventions supported housing needs and connected youth with landlord resources and housing workers who help walk youth through the process of renting accommodations, and life skills programming (e.g., laundry, groceries, etc.) to set participants up for success with independent living. YWS also has an “after-care” program in place to support clients who are no longer residing at the shelter. This program helps youth with concerns about landlords or roommate mediation.

#### Financial

Financial stressors (*n* = 5) such as difficulty affording medical expenses (e.g., ambulance bills, medications), school, clothing, food, housing, applying for citizenship, and means to stay in touch with loved ones (e.g., calling cards, SIM cards, data plans) contributed negatively to mental health. One client described their internal conflicts between being encouraged by YWS to pursue their education and working to afford a citizenship application or send money back home to their family: “I already start to think about to work, my family needs some money, citizenship, like, I can do it anytime, and they [YWS] asked me to go to school” (YP B6). YWS provided support with financial matters and basic material needs in the form of providing clothes, shoes, and food, as well as assisting them with system navigation, connecting to resources and applying for financial assistance such as ODSP.

#### Education

While youth were academically driven and valued education, multiple barriers to education (*n* = 6) for homeless refugee youth existed such as international student fees, difficulty accessing OSAP, lengthy periods of waiting for study permits, and language barriers.

The opportunity to attend school related to positive mental health “Iam very happy with attending school. I am less stressed, less depressed” (YP B4). However, financial accessibility remained a significant barrier with one youth describing “I get to Canada, I can work and go back to school, but the school fees were a bit, very large for me” (YP B1). Legal and administrative processes represented another hurdle to access education. The lengthy periods of time to obtain study permits caused substantial stress, boredom and frustration throughout the process as one participant described the “(study permit). is causing me stress. If I got that one (study permit), I would go back [to school], I would go back to the upgrading” (YP B1). Language fluency, and comfort and confidence studying in English posed as additional barriers to the education, with one refugee youth explaining: “I was worried that it would be very difficult for me to learn in a different language and it was and still is” (YP B4).

YWS supported youth by promoting the value of education, encouraging school attendance, providing financial support for textbooks and tutors, assisting with system navigation and accessing available opportunities. As youth noted, “they help me in the shelter. I have a case worker. Like I did not know I could study for free” (YP B2).

#### Employment

Youth valued the prospect of employment for several reasons, including its affording a source of income, transferrable skills, structure to occupy their days, career objectives and a sense of purpose. Barriers to employment (*n* = 6) for youth were prominent with issues obtaining work permits, lack of work experience in Canada, language proficiency, and the competing interest of furthering their education. The process for obtaining work permits was lengthy, with one participant describing their advocacy at the city level for shorter turnaround times: “I asked him (the mayor) if the government can just make it, like, maybe to be like two weeks, three weeks, I asked him. You know? Someone cannot wait, like, six, five months, it is really bad” (YP B5). Other stressors include the tension between working and attending school where youth must often choose between one or the other as this participant describes “actually, I still go to work, but now I cannot do because I am still in school” (YP B6).

#### Sense of Safety

The sense of safety (*n* = 4) experienced at YWS was essential to success in Canada, with this youth outlining how they felt “relieved and relaxed not having to be careful about everything” (YP B5) and “better chances of you having good stable mental health coming down here (to Canada)” (YP B5). Another participant echoed the sense of safety they felt just by being in Canada: “my country, like it is not like really safe… they just kill you for nothing… I really happy like to, come here to, like, Canada” (YP B6). Canada was described as a safe haven by youth from minority backgrounds reinforcing the need for security: “in (COUNTRY), the incident that would make me come to Canada now… in (COUNTRY) they do not accommodate, anything like bisexual, homosexual” (YP B1). YWS conceptualized safety and stability as first-line mental health interventions and achieved this by ensuring consistency and predictability at the shelter.

### Acculturative Stress, a Product of Transition to Canada

Youth described experiencing acculturative stress as a result of needing to adjust to new social and cultural norms, operate in the English language, and navigate a foreign system.

#### Culture Shock

Several youths (*n* = 3) described experiencing culture shock in their transition to Canada and spoke to their struggle with orienting themselves to a new language, social norms and expectations, customs, and social systems. Recounting the overwhelming nature of this culture shock, one youth said “(the rules of Canada), what I have to do, what I cannot do. How can I use the train, the buses, the streets I need like, I just get my driver license; I did not know how to apply for it. I do not know anything.” (YP B2). Others described experiencing “culture shock in a good way” (YP B5) as they no longer had to worry about their safety as in the home country.

For some, culture shock involved adjusting to North American seasonal manners of dress: “I came here during summer going into fall. In terms of, like, dressing, it was, like, strange, you know? Like I had to adapt to it” (YP B5). Cold weather was an environmental form of culture shock, with youth exclaiming that “the cold is difficult” (YP B1) and “the only thing I did not like was the cold” (YP B6). Participants further described the steep learning curve for adjusting to the cold weather: “the first day it was snowing, I came outside, I did not even feel cold I was feeling so cold like why did not I wear referring to coat” (YP B5). However, the same participant also framed this environmental culture shock positively and expressed their desire to immerse into Canadian culture: “Yeah I would like to learn to play hockey still. And then make snow balls” (YP B5).

#### Language

Operating in an unfamiliar language (*n* = 4) created stress that youth described as influencing their wellbeing. This participant opined that their English fluency affected society's perception of them: “if you do not speak English, it is like you do not know nothing” (YP B6). Canada having two official languages created further issues and some made decisions as to where to live based on language spoken. This participant described their reasoning for moving from Montreal to Toronto as “they speak French in Montreal, everything in French, street, name of the station, and it is so hard for you” (YP B2). YWS supported youth in the development of their language skills by connecting them with ESL programs or tutors.

### Fostering a Sense of Control

A sense of control was central to fostering resilience among youth. A plethora of system-level factors that affected the mental health of refugee youth are described earlier. Participants combatted these factors through individual-level actions in the form of goal-directedness. Independence and responsibility were also displayed by participants and cultivated at the community-level by YWS to create a sense of ownership over youth choices and actions.

#### Goal-Directedness

Participants demonstrated strong future orientation, control, empowerment and goal-directedness (*n* = 5). Goal-directedness was a protective trait in affording youth meaning, direction and purpose, while focusing on factors outside of participants' control manifested as anxiety and stress.

Some participants explicitly identified their goals for the future as contributing to their sense of control: “I want to go back to school. Try to study hard. Be a better person” (YP B1). In addition to appreciating the intrinsic value of education, education was also understood as a steppingstone to employment and financial independence. In explaining their rationale for pursuing further schooling, one youth stated “I choose to make my own decision. I say Iam going to finish school and after Iam going to try to find a good job” (YP B3).

Oftentimes, goals and ambitions imbued youth with hope and determination; conversely, this future-orientation also manifested itself as anxiety or stress. This maladaptive coping was driven by uncertainty and an inability to anticipate the future, as underscored by this participant describing the status of asylum claims: “(always thinking about it. If they do not accept me here what I should do?)” (YP B2). Furthermore, this sense of anxiety was informed by feelings of powerlessness and lack of control: “oh you have to do something but it is not in my hand” (YP B2). Stress similarly drove future orientation and fostered resilience for many. The same participant exemplified this phenomenon and described their worries as motivating factors: “Iam so worried if I do not finish my school” and “always thinking about my future. What Iam going to do? What Iam going to work?” (YP B2).

In contrast to many of their peers who demonstrated impressive resilience in the face of significant adversity and uncertainty, one youth conveyed a sense of hopelessness over the abrupt separation from her family. The youth described “I was very sad and stressed because I was thinking of my family”, explaining that they felt sad “a lot because of my family's situation, but I had no one to talk to then” (YP B4).

#### Independence and Responsibility

Heightened levels of independence and responsibility (*n* = 4) often cultivated a sense of control. One youth spoke fondly of their newfound responsibility: “(even chores made me feel like I was doing something good and I felt independent and it gave me a sense of ownership)” (YP B4). The change in youth's levels of independence was perceived differently depending on whether they welcomed or were overwhelmed by new responsibilities. Some youth developed increased worry due to a heightened level of responsibility and self-reliance post-migration: “I have a responsibility here. Before I have nothing to worry, I have to do all by myself” (YP B2).

### Social Connectedness and Belonging

Social connectedness and belonging were pivotal to fostering resiliency and YWS played a central role in nurturing this welcoming environment. Participant narratives clearly delineated the importance of contributions of family and friends to youth's mental health.

#### Family

Family relationships (*n* = 5) were neither universally positive nor negative; they appeared to contribute to both resilience and stress, depending on the unique relationship dynamics and contextual factors at play. Some youth described emotionally supportive family relationships as bolstering their resiliency: “when I talk to my mom, I talk to her like, ‘Mom, Iam stressed,’ and then she feels that talking to me to just tell me take my time and stuff like that, I hang up, I just feel good” (YP B5). Unfortunately, other participant experiences proved to be a source of stress because of separation from family, supporting them financially, meeting their expectations, worrying about their safety, and helping other family members migrate to Canada. Additionally, selected family relationships were strained due to histories of abuse, or due to rejection for their sexual orientation.

#### Peers

The quality and quantity of support youth derived from peer relationships was, similarly, also variable. Some friendships encouraged resilience as one participant reported “(my mental health was not in a good state, but it changed because there are a lot of people here that help me: my friends, boyfriend and staff)” (YP B4). Moreover, youth seemed to derive particular strength from friends with whom they had shared lived experiences, such as a common country of origin, language, or religion, as this youth discussed: “we went to high school back together in (COUNTRY). So, every time we talk, I feel like, you know, we were still back home, I feel, like, blessed having them there.” (YP B5).

Another participant explained that when they felt stressed, they liked “talking to people for fun” (YP B5). Socializing with friends was integral to resilience irrespective of whether this youth confided in others about their struggles: “they do not even know if Iam stressed or nothing because I do not make it known” (YP B5). This preference for privacy in dealing with personal challenges was echoed by others, as illustrated by this youth: “I do not like talk about my thing worries, it is my personality” (YP B3). Others expressed a desire to be open and described overcoming shyness and leaning on others for support: “I am a very shy person which makes it hard for me to express myself and open up to people. I do not think it is good because it can add on to your stress and it is good to talk to people” (YP B4).

Several youths described also having smaller social networks and being more socially isolated since arriving in Canada: “I have no friends here yet” (YP B2). Compounding this, some faced difficulty staying in touch with friends back home as they did not have calling cards or lost contact information. Despite this, having a fewer number of friends in Canada was not always a concern, as this participant stated unreservedly with respect to their social supports that “just my mom, it is ok for me” (YP B1). Contrastingly, others struggled significantly with the social isolation and shared “I had no one to talk to… I would just sit alone and cry” (YP B4).

#### Sense of Community

The community (*n* = 4) nurtured resilience, with multiple youth specifically referencing YWS with its welcoming physical space, programming, and engaged and devoted staff. Youth described experiencing staff's welcoming nature from the outset of their YWS experiences. As this participant described, “before I came here to YWS, and I call on the phone, and they [the staff] already show me, like they talk to me, I already see they are like a good person” (YP B3). Youth's experiences with staff and the YWS community were characterized by kindness, guidance, caring, love and support, with participants reflecting that “they show me love” and “they care about everyone” (YP B3). Youth themselves further initiated community by watching sport “together... not everyone likes soccer still. They like basketball and some people watch hockey, we try to create space for each other” (YP B5). Outside of YWS, other community agencies supporting youth were identified. One youth spoke to a LGBTQ organization and described their programming as proactive in making connections: “you do not even have to come to them, they will come to you” (YP B1).

Interestingly, some youth described feeling cared for by the Canadian government and broader community: “Canada was really good place to me” (YP B6). Expressing a similar sentiment, another participant stated: “the shelter [is] caring, they are trying, government itself is trying” (YP B1). Meanwhile, others described a lack of warmth and community-feeling in Toronto that contributed feelings of social isolation: “(in Toronto) nobody even care about what you do, you know? They just walk by you.” (YP B5).

## Discussion

In recent years, YWS has seen an emergence of refugee youth represented in their clientele. This increasing subset of the YWS population requires specific attention as their issues are unique to their lived experience. It is clear there are specific post-migratory mental health issues that arise and unique mechanism of resilience that are prevalent in this population.

Participants specifically outlined the educational and work opportunities in Canada as a major cause for migration. However, youth in our study described post-migratory system-based concerns with accessing education, employment and the immigration process as key to propagating their mental health issues. Our findings were similar to Shakya et al. (15) where their study of Toronto-based refugee youth demonstrated educational aspirations being strengthened post-migration. The tension of a refugee youth's educational ambitions being usurped by the more immediate needs of shelter, housing and food security was also noted (15). Our study further described that the additional roles of refugee youth, including acting as service navigators and breadwinners, detracted from their personal goals. System-based issues created tremendous responsibilities and aligned with previous work by McFarlane et al. (16) which emphasized concerns of early parentification and in our study, impacted mental health due to an inability to meet goals of education and employment.

Other findings of post-migratory system-based factors that impacted refugee youth mental health related to housing insecurity, finances and sense of safety. Our findings were in keeping with the broader understanding of Miller and Rasmussen (9), who posited that displacement stressors impacted refugee mental health by creating a sense of lack of control in relation to these factors. Further, others suggested that uncertainty, lack of control, passivity, and feelings of meaninglessness and powerlessness are linked to psychosocial distress and suffering, and accelerated asylum processes and engagement with empowering activities would contribute positively to refugees' mental wellbeing (17). Bjertrup et al.'s (17) work emphasized that refugee youth have a hierarchy of mental health needs, and that meeting basic needs promotes positive mental health and should be conceptualized as a mental health intervention as at YWS.

Our study also showed that acculturative stress including culture shock and language impact mental health. Acculturative stress was often found to be overlooked in contemporary literature and noted to impact the socioeconomic development of refugee youth strongly (18). Other work also outlines that issues of language barriers, cultural differences, discrimination, racism and bullying affect social connectedness and mental health (16, 19).

Refugee youth in our study additionally emphasized goal directedness, independence and responsibility and social connectedness as key factors in resiliency. These findings paralleled previous studies which demonstrated that valuing education and school attendance in addition to fostering agency, self-determination and empowerment among refugee youth facilitated their post-migration resiliency (20). Likewise, a supportive and positive family life, a sense belonging, and perceived community support appeared to promote resilience among refugee youth (21). Trusting relationships with adults and peers, as well as prosocial affiliations, possibly contributed meaningfully to youth's resilience (22).

The outcomes of our study paired with previous literature inform a model whereby future interventions catered toward refugee youth mental health can be designed. As our results reflected, refugee youth indicated that basic needs such as the opportunity to have housing security was of utmost importance. Additionally, Maslow's Theory of Human Motivation, a hierarchy where the basic needs of safety and security usually predominate the development of and engagement in higher order needs (23). As such, it follows that an intervention that targeted and prioritized housing security including resolutions for precarious housing and homelessness including emergency shelter in addition to long-term placement is imperative and rooted in our evidence. A phased model where the primary focus is on programming targeting the basic and immediate needs of safety and security reflects other interventions in addition to the theoretical basis (24). Progressively and guided by the youth, a proposed intervention could then be geared toward goals that require more significant investment in addition to time to accomplish. Examples of such long-term goals such as employment, education, accommodation, and immigration status should also be incorporated (24). Refugee youth with immigration experiences will require time to integrate into Canadian society. Through time after immediate needs are met and programs should be more attentive to their mental health needs. As reflected and our study population demonstrated, youth gained experience in accessing mental health in Canada after 1–2 years living in the country. Youth were only able to attend to this after their immediate needs were met. Furthermore, as described earlier, their more immediate needs would be necessary to attend to prior to mental health needs. Our proposed model echoes the stance of previous work which indicates that refugees' hierarchy of needs during resettlement likely means. Issues of immediate safety and survival take precedence over reflections of their experiences in settings such as psychotherapy (11). Once developed, this work could be used to optimize care in the emergent and longitudinal phase for a population experiencing multiple intersections of marginalization.

### Strengths

Our work offered detailed perspectives into the lived experience of refugee youth experiencing homelessness in Canada; to our knowledge, this population had not previously been qualitatively studied. This study outlined key factors that impacted mental health and fostered resiliency in a vulnerable population. We also offered a model to inform future interventions based on our results and previous literature by which the needs of refugee youth experiencing homelessness could most effectively be addressed.

### Limitations

A main limitation of this study was the small sample size of six youth, who were predominantly older (> 18 years old), were unaccompanied by family and were refugee claimants. Findings were also drawn from one point-in-time interview and hence, long-term perspectives about YWS could not be elucidated. All study participants also identified as homeless youth and were already accessing emergency shelter or transitional housing services at YWS. Although these youth offered their experiences prior to engagement with YWS, findings could differ in populations who were fully unsheltered, insecurely housed or provisionally accommodated in other settings (25). This study further explored the experiences of refugee youth who resettled in Canada, a high-income country as opposed to a low- or middle-income country and the generalizability here may be limited due to different stressors.

## Conclusions

The present study offers a holistic approach to understanding factors impacting the mental health and resiliency of refugee youth residing at a shelter and referral agency. A model for planning future interventions was proposed and was informed by the lived experience of participants. This model focuses on initially on immediate and basic needs and reflected evidence gathered through this work to attend to long-term needs once individuals had integrated into society. Future efforts will be directed toward knowledge translation, with a focus on practice guidelines and clinical implications.

## Data Availability Statement

The original contributions presented in the study are included in the article/Supplementary Material, further inquiries can be directed to the corresponding author.

## Ethics Statement

The studies involving human participants were reviewed and approved by University of Toronto Research Ethics Board. Written informed consent to participate in this study was provided by the participants' legal guardian/next of kin.

## Author Contributions

BK contributed to the data analysis, data interpretation, and manuscript preparation. JW contributed to the data analysis and manuscript preparation. MP contributed to the study conception, design and conduction, and was also engaged in the data analysis and interpretation phase in addition to manuscript preparation. All authors contributed to the article and approved the submitted version.

## Conflict of Interest

The authors declare that the research was conducted in the absence of any commercial or financial relationships that could be construed as a potential conflict of interest.

## Publisher's Note

All claims expressed in this article are solely those of the authors and do not necessarily represent those of their affiliated organizations, or those of the publisher, the editors and the reviewers. Any product that may be evaluated in this article, or claim that may be made by its manufacturer, is not guaranteed or endorsed by the publisher.
