# Supplementary material for: Perspectives of Refugee Youth Experiencing Homelessness: A Qualitative Study of Factors Impacting Mental Health and Resilience
Source: Front Psychiatry. 2022 Jun 6;13:917200. doi: 10.3389/fpsyt.2022.917200 (PMC9211750; doi:10.3389/fpsyt.2022.917200)
Supplement: Supplementary file 1 [file Data_Sheet_1.docx]

**Supplemental Material**

**Appendix A. Qualitative Interview Guide (Youth Participants)**

I would like to thank you again for your willingness to participate in our study. I would also like to remind you to let me know if there are any questions that you would prefer not to answer, or any answers that you are uncomfortable with and would like to have removed from the record.

The purpose of our research study is to understand what contributes to stress and resilience in young people who are refugees and living in a shelter.

1. Baseline information
   1. How old are you?
   2. How would you identify your gender?
   3. What languages do you speak?
   4. In which country were you born? From which country is your family?
   5. Do you feel comfortable telling me why you left _____________?
      1. If yes: Why did you leave ________________?
   6. Can you walk me through your journey before you landed in Canada?
      1. Did you live in any countries for more than one month before landing in Canada? If so, which countries and for how long?
   7. When did you land in Canada? / How long have you been in Canada?
   8. Df yes: eel comfortable tells use why you left _____________? in)other youthher youth at the At what legal stage is your refugee claim right now?
2. Mental health
   1. Is mental health important to you? Please explain.
   2. What do you think of your own mental health or emotional health?
   3. Have you been diagnosed with a mental health disorder or illness?
   4. Are you receiving support for your mental health? Are you receiving any specific treatment for your mental health?
   5. How do you deal with the things that bother you? Who do you reach out to for support?
      1. Are there things or qualities in yourself that make your mental health better or worse?
      2. Are there things about your family that make your mental health better or worse?
      3. Are there things about your community (the shelter, your school, your work, your friends) that make your mental health better or worse?
      4. Are there things about living in Canada that make your mental health better or worse?
3. Resiliency
   1. You’ve been through a lot in your life. How do you think you’ve gotten through everything so far?
   2. What makes you stronger?
      1. What about yourself makes you stronger?
      2. What about your family makes you stronger?
      3. What about your community (the shelter, your school, your work, your friends) makes you stronger?
      4. What about living in Canada makes you stronger?
   3. How do you fill your days? Are you in school? Do you have a job?
   4. You said that you were involved in ______________. What has helped you be successful?
   5. You said that ______________ has helped you be successful. How was it helped you? Why do think this is helpful? Can you think of anything else that could help you be more successful?
4. Final comments
   1. Do you have anything additional comments you would like to add?
